# Poverty and Cancer Disparities in Ohio

**DOI:** 10.5888/pcd15.180332

**Published:** 2018-12-06

**Authors:** John Kollman, Holly L. Sobotka

**Affiliations:** 1 Office of Health Improvement and Wellness, Ohio Department of Health, Columbus, Ohio

## Abstract

**Introduction:**

Poverty is associated with higher cancer rates, cancer risk factors such as tobacco use and obesity, and lack of access to cancer screening and treatment. This analysis examined differences in cancer outcomes and associated factors among the poorest counties and the most affluent counties in Ohio.

**Methods:**

We compared cancer incidence and mortality rates and prevalence of selected cancer risk factors between the 12 poorest counties in Ohio and the 10 most affluent counties in Ohio from January 1, 2011, through December 31, 2015. We also compared stage at diagnosis of selected cancers and the health insurance and treatment status of people with cancer.

**Results:**

The mortality rate for all cancers combined was 19% higher in the poorest counties (192.2 per 100,000) than in the most affluent counties (161.9 per 100,000). Cervical cancer and other smoking-related cancers had higher rates in the poorest counties, where they were more likely to be diagnosed at a late stage. The prevalence was significantly higher in Ohio’s poorest counties for current tobacco smoking (25.6% vs 17.1%), obesity (32.7% vs 28.3%), and physical inactivity (29.7% vs 23.0%). Among people with cancer, a smaller percentage had private health insurance (42.9% vs 33.0%) and a greater percentage had no treatment (8.9% vs 10.4%) in the poorest counties.

**Conclusion:**

This study demonstrates disparities in cancer incidence, mortality, and stage, and differences in cancer risk factors, health insurance, and treatment status between Ohio’s poorest and most affluent counties. This information may help to target public health interventions for the prevention, early detection, and control of cancer.

## Introduction

Cancer affects all population groups in the United States; however, certain groups bear a disproportionate burden of cancer. Cancer-related disparities are differences between groups of people in cancer incidence, mortality, and stage or in risk factors associated with cancer, such as tobacco use or lack of access to cancer screening. People with lower socioeconomic status have higher cancer death rates than those with higher socioeconomic status, regardless of demographic factors such as race/ethnicity (1). The National Cancer Institute reports that many factors can cause cancer disparities, including poverty and a resultant lack of high-quality medical care (2).

The relationship between socioeconomic status and cancer incidence and mortality in the United States is well established (3–5), and it varies according to cancer site or type (5). Cancers associated with poor areas in the United States include cancers of the lung and bronchus, colon and rectum, cervix, oral cavity and pharynx, and liver and intrahepatic bile duct (3). Cancers associated with more affluent areas in the United States include cancers of the breast, prostate, and thyroid and melanoma of the skin (3). One study using US data found that rates for some cancers differed by as much as a factor of 2 between the poorest groups and the most affluent groups (3). Residents of poor areas are also more likely than residents of wealthier areas to receive a diagnosis cancer at a late stage (4).

We examined cancer disparities in Ohio to determine whether the relationship between poverty and cancer was apparent at the county level. Ohio is suitable for such an analysis because the state’s poverty rates vary by county, ranging from 4.5% to 33.0%, such that poor counties are mostly in the Appalachian region of Ohio and the more affluent counties are mostly suburban and do not include metropolitan areas. The objective of this study was to describe differences in cancer incidence and mortality rates, stage at diagnosis, cancer risk factors, health insurance status, and treatment status between the poorest counties and the most affluent counties in Ohio.

## Methods

We studied populations in the 12 poorest and the 10 most affluent counties in Ohio. These counties were identified by the percentage of the county population living in poverty in the 2011–2015 American Community Survey (6). The US Census Bureau uses a set of income thresholds that vary by family size and composition to determine who is living in poverty. In 2015, for example, the minimum family income threshold for poverty was $12,331 for a person younger than 65 and $24,036 for a family of 4 (2 adults and 2 children aged <18 y) (7). Twelve counties in Ohio had poverty rates of 20% or more in 2011–2015 (Adams, Ashtabula, Athens, Gallia, Highland, Jackson, Lucas, Meigs, Morgan, Pike, Scioto, and Vinton) and were defined as the poorest counties (8). All 12 counties, except Lucas County, are in the Appalachian region. The population in this group of counties was 904,834 in 2010, 7.8% of the Ohio population, and approximately 82.6% non-Hispanic white and 10.2% non-Hispanic black. Ten counties had poverty rates of less than 10% in 2011–2015 (Auglaize, Delaware, Geauga, Lake, Madison, Medina, Mercer, Putnam, Union, and Warren) and were defined as the most affluent counties (8). Most of these counties are adjacent to metropolitan areas. The population in this group of counties was 1,099,666 in 2010, 9.5% of the Ohio population, and approximately 91.8% were non-Hispanic white and 2.5% were non-Hispanic black.

### Data sources

We obtained data on cancer incidence, stage at diagnosis, insurance status, and summary treatment status from the Ohio Cancer Incidence Surveillance System (OCISS), the central cancer registry for Ohio (9). We coded cancer cases to the *International Classification of Diseases for Oncology, Third Edition* (ICD-O-3), and we categorized data on 23 sites and types of cancer according to the conventions of the National Cancer Institute Surveillance, Epidemiology, and End Results (SEER) Program (10,11). We tabulated data on incidence from cancer cases diagnosed from January 1, 2011, through December 31, 2015, and accessed through OCISS in January 2018. We calculated cancer incidence rates by counting only invasive cancer cases; we excluded in situ tumors except for cases of in situ bladder cancer. We selected cancers for which the mortality rate in the poorest counties was significantly higher than the mortality rate in the most affluent counties; for these cancers, we calculated the percentage of cases diagnosed at a late stage in the poorest counties and in the most affluent counties. We classified stage at diagnosis by using SEER Summary Stage 2000 and the following categories: early stage (in situ and local), late stage (regional and distant), and unstaged/missing (insufficient information was available to determine the stage of disease at the time of diagnosis or the case was reported without information on stage) (12). Insurance status was based on data for primary payer at diagnosis (“Primary Payer at DX”), and treatment status was based on a summary measure of all treatment modalities (“Rx Summ-Treatment Status”), categorized into the following groups: no treatment given, treatment given, active surveillance (watchful waiting), and unknown.

We obtained cancer mortality data from the Ohio Bureau of Vital Statistics at the Ohio Department of Health (13). These data, which indicate underlying cause of death, were coded by using the *International Statistical Classification of Diseases and Related Health Problems, 10th Revision* (ICD-10) (14) and tabulated for 23 cancers according to methods outlined in the SEER Program’s Cause of Death Recode (15).

We used data from the Ohio Behavioral Risk Factor Surveillance System (BRFSS) for 2011–2015 to analyze 5 risk factors (16): current smoking (smoking ≥100 cigarettes in lifetime and currently smoke cigarettes every day or some days), obesity (a body mass index of ≥30.0 [weight in kilograms divided by height in meters squared]), physical inactivity (no physical activity or exercise other than regular job during the past 30 days), heavy drinking (men having >2 drinks per day and women having >1 drink per day), and a Papanicolaou (Pap) test in the previous 3 years among women aged 21 to 65 (data on this variable not collected in 2013 BRFSS). We determined the prevalence of each risk factor in the poorest counties and in the most affluent counties. The BRFSS is an annual telephone survey conducted by the Ohio Department of Health and supported by the Centers for Disease Control and Prevention (CDC) and is the primary source of health information on Ohio residents aged 18 years or older.

### Statistical analyses

We tabulated data on incidence and mortality rates per 100,000 people and age-adjusted these data to the 2000 US standard population by using 19 five-year age groups (<1 y, 1–4 y, 5–9 y, . . . ≥85 y) (17). We calculated rate ratios (rate among the poorest group divided by the rate among the most affluent group) for each cancer site, along with the 95% confidence intervals of the rate ratios, which were used to determine significant differences. If the 95% confidence interval did not contain 1.0, we concluded that a significant difference between the 2 groups of counties existed at the .05 significance level. We analyzed data by using the Ohio Public Health Data Warehouse and SAS version 9.4 for Windows (SAS Institute Inc). To test for differences in stage at diagnosis, insurance status, treatment status, and risk factors, we used the 2-proportion *z* test at the .05 significance level.

## Results

Approximately 320,000 new invasive cancer cases were diagnosed in Ohio from 2011 through 2015; 24,588 cases were in the 12 poorest counties and 30,229 cases were in the 10 most affluent counties. During the same period, more than 126,000 deaths in Ohio were caused by cancer: 10,319 cancer deaths in the poorest counties and 10,571 cancer deaths in the most affluent counties.

### Cancer incidence

**Figure 1:**
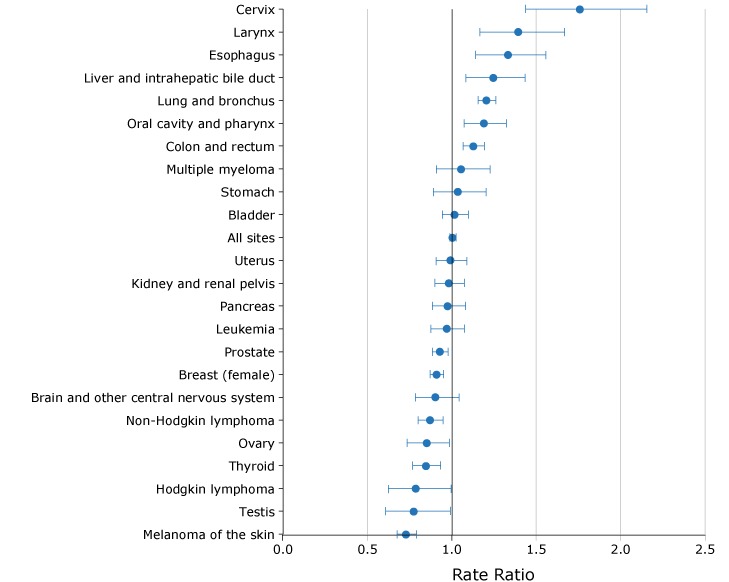
Ratios comparing cancer incidence rates in the 12 poorest counties with cancer incidence rates in the 10 most affluent counties in Ohio, by site or type of cancer, 2011–2015. Rates are per 100,000, age-adjusted to the 2000 US standard population and sex-specific for breast, cervix, ovary, prostate, testis, and uterus. Types of cancer were categorized according to the conventions of the National Cancer Institute Surveillance, Epidemiology, and End Results (SEER) Program (11). Source of data on incidence: Ohio Department of Health (9). **Table:** 

The incidence rate of all cancers combined was 464.0 per 100,000 in the poorest counties and 461.3 per 100,000 in the most affluent counties. The incidence rate was significantly higher in the poorest counties than in the most affluent counties for the following cancers: cervix (9.5 vs 5.4 per 100,000 women), larynx (4.6 vs 3.3 per 100,000), esophagus (6.0 vs 4.5 per 100,000), liver and intrahepatic bile duct (7.1 vs 5.7 per 100,000), lung and bronchus (74.9 vs 62.1 per 100,000), oral cavity and pharynx (12.5 vs 10.5 per 100,000), and colon and rectum (44.9 vs 39.8 per 100,000) (Figure 1). The most affluent counties had significantly higher incidence rates for melanoma of the skin, thyroid cancer, non-Hodgkin lymphoma, ovarian cancer, female breast cancer and prostate cancer. The incidence rate for cervical cancer in the poorest counties was 1.8 times higher than the rate in the most affluent counties.

Site or Type of Cancer	Rate Ratio (95% Confidence Interval)
Cervix	1.8 (1.44–2.16)
Larynx	1.4 (1.16–1.67)
Esophagus	1.3 (1.14–1.56)
Liver and intrahepatic bile duct	1.2 (1.08–1.43)
Lung and bronchus	1.2 (1.15–1.26)
Oral cavity and pharynx	1.2 (1.07–1.32)
Colon and rectum	1.1 (1.07–1.19)
Multiple myeloma	1.1 (0.91–1.22)
Stomach	1.0 (0.89–1.20)
Bladder	1.0 (0.94–1.10)
All sites	1.0 (0.99–1.02)
Uterus	1.0 (0.91–1.09)
Kidney and renal pelvis	1.0 (0.90–1.07)
Pancreas	1.0 (0.88–1.08)
Leukemia	1.0 (0.88–1.07)
Prostate	0.9 (0.88–0.98)
Breast (female)	0.9 (0.87–0.95)
Brain and other central nervous system	0.9 (0.78–1.04)
Non-Hodgkin lymphoma	0.9 (0.80–0.95)
Ovary	0.9 (0.74–0.99)
Thyroid	0.8 (0.77–0.93)
Hodgkin lymphoma	0.8 (0.62–1.00)
Testis	0.8 (0.61–0.99)
Melanoma of the skin	0.7 (0.67–0.79)

### Cancer mortality

**Figure 2:**
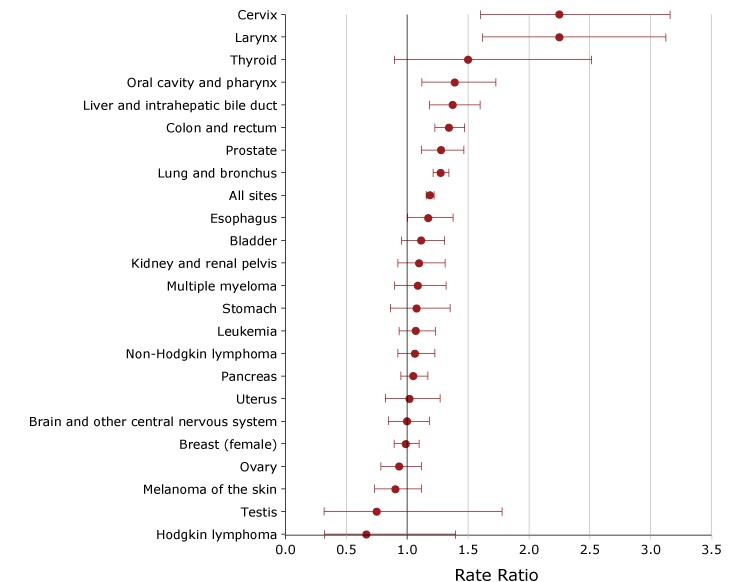
Ratios comparing cancer mortality rates in the 12 poorest counties with cancer mortality rates in the 10 most affluent counties in Ohio, by site or type of cancer, 2011–2015. Rates are per 100,000, age-adjusted to the 2000 US standard population and sex-specific for breast, cervix, ovary, prostate, testis, and uterus. Types of cancer were categorized according to the conventions of the National Cancer Institute Surveillance, Epidemiology, and End Results (SEER) Program (11). Source of data on mortality: Ohio Department of Health (13). **Table:** 

The mortality rate for all cancers combined was 19% higher in the poorest counties (192.2 per 100,000) than in the most affluent counties (161.9 per 100,000). In addition, the mortality rate was significantly higher in the poorest counties for the following sites or types of cancers: larynx (1.8 vs 0.8 per 100,000), cervix (3.6 vs 1.6 per 100,000 women, oral cavity and pharynx (3.2 vs 2.3 per 100,000), liver and intrahepatic bile duct (6.6 vs 4.8 per 100,000), colon and rectum (18.0 vs 13.4 per 100,000), prostate (20.5 vs 16.0 per 100,000 men), and lung and bronchus (56.9 vs 44.6 per 100,000) (Figure 2). The greatest difference in mortality between the poorest counties and the most affluent counties was for cervical cancer and laryngeal cancer. The cervical cancer mortality rate in the poorest counties was 2.3 times the rate in the most affluent counties. The laryngeal cancer mortality rate in the poorest counties was 2.3 times the rate in the most affluent counties. We found no cancers for which the mortality rate was significantly higher in the most affluent group.

Site or Type of Cancer	Rate Ratio (95% Confidence Interval)
Larynx	2.3 (1.6–3.1)
Cervix	2.3 (1.6–3.2)
Thyroid	1.5 (0.9–2.5)
Oral cavity and pharynx	1.4 (1.1–1.7)
Liver and intrahepatic bile duct	1.4 (1.2–1.6)
Colon and rectum	1.3 (1.2–1.5)
Prostate	1.3 (1.1–1.5)
Lung and bronchus	1.3 (1.2–1.3)
All sites	1.2 (1.2–1.2)
Esophagus	1.2 (1.0–1.4)
Bladder	1.1 (1.0–1.3)
Kidney and renal pelvis	1.1 (0.9–1.3)
Multiple myeloma	1.1 (0.9–1.3)
Stomach	1.1 (0.9–1.4)
Leukemia	1.1 (0.9–1.2)
Non-Hodgkin lymphoma	1.1 (0.9–1.2)
Pancreas	1.1 (0.9–1.2)
Uterus	1.0 (0.8–1.3)
Brain and other central nervous system	1.0 (0.8–1.2)
Breast (female)	1.0 (0.9–1.1)
Ovary	0.9 (0.8–1.1)
Melanoma of the skin	0.9 (0.7–1.1)
Testis	0.8 (0.3–1.8)
Hodgkin lymphoma	0.7 (0.3–1.4)

### Stage at diagnosis

A greater percentage of several cancers (cervix, colon and rectum, larynx, oral cavity and pharynx, lung and bronchus, and all cancers combined) was diagnosed at a late stage in the poorest counties than in the most affluent counties (Table 1). The percentage of cervical cancers diagnosed at a late stage was significantly different between the 2 types of counties: 60.0% in the poorest counties and 45.1% in the most affluent counties. We found a significantly higher percentage in the poorest counties than in the most affluent counties of cases that were unstaged or had missing information on stage for lung and bronchus cancer and all cancers combined.

**Table 1:** Percentage of Cases of Selected Cancers,^a^ by Stage at Diagnosis,^b^ in the 12 Poorest Counties and 10 Most Affluent Counties in Ohio, 2011–2015^c^

Primary Cancer Site or Type, by Stage at Diagnosis	% (95% CI)		*P* Value^d^
	Poorest Counties	Most Affluent Counties	
**Late-stage diagnosis (regional and distant)**			
All cancer sites and types	43.5 (42.9–44.1)	40.5 (40.0–41.0)	<.001
Cervix	60.0 (53.2–66.5)	45.1 (37.2–53.1)	.004
Colon and rectum	54.6 (52.6–56.6)	52.9 (51.0–54.8)	.22
Larynx	45.4 (39.2–51.6)	38.7 (32.7–45.0)	.13
Liver and intrahepatic bile duct	40.5 (35.6–45.5)	45.7 (40.7–50.8)	.14
Lung and bronchus	71.3 (69.8–72.6)	70.9 (69.5–72.3)	.72
Oral cavity and pharynx	66.6 (62.9–70.1)	65.6 (62.0–69.1)	.70
Prostate	19.2 (17.8–20.8)	19.4 (18.1–20.7)	.85
**Unstaged or missing information on stage^e^**			
All cancer sites and types	9.0 (8.7–9.4)	8.1 (7.8–8.4)	<.001
Cervix	5.5 (2.9–9.3)	6.8 (3.4–11.8)	.59
Colon and rectum	6.5 (5.6–7.5)	7.5 (6.5–8.6)	.15
Larynx	5.8 (3.3–9.3)	6.3 (3.7–10.1)	.79
Liver and intrahepatic bile duct	22.4 (18.4–26.8)	19.9 (16.0–24.2)	.39
Lung and bronchus	10.6 (9.6–11.6)	8.8 (8.0–9.7)	.007
Oral cavity and pharynx	5.0 (3.5–6.9)	4.7 (3.3–6.5)	.79
Prostate	8.1 (7.1–9.2)	9.2 (8.3–10.2)	.15

Abbreviation: CI, confidence interval.

a Cancers were selected because the mortality rate for these cancers in the poorest counties was significantly higher than the mortality rate for these cancers in the most affluent counties.

b Source of data for stage at diagnosis: Ohio Cancer Incidence Surveillance System, Ohio Department of Health (9). Data were classified by using SEER Summary Staging Manual 2000 (12).

c Identified by the percentage of the county population living in poverty in the 2011–2015 American Community Survey (6). Twelve counties in Ohio had poverty rates of 20% or more in 2011–2015 (Adams, Ashtabula, Athens, Gallia, Highland, Jackson, Lucas, Meigs, Morgan, Pike, Scioto, and Vinton). Ten counties had poverty rates of less than 10% in 2011–2015 (Auglaize, Delaware, Geauga, Lake, Madison, Medina, Mercer, Putnam, Union, and Warren).

d Differences in percentages between poorest counties and most affluent counties determined by the 2-proportion *z *test; significance set at <.05.

e Insufficient information was available to determine the stage of disease at the time of diagnosis or the case was reported without information on stage.

### Cancer risk factors

The prevalence of current smoking, obesity, and physical inactivity was significantly higher in the poorest counties than in the most affluent counties (Table 2): 25.6% versus 17.1% for current smoking, 32.7% versus 28.3% for obesity, and 29.7% versus 23.0% for physical inactivity. The prevalence of heavy drinking in the poorest counties (5.6%) was similar to the prevalence in the most affluent counties (5.5%). During 2011–2015 (excluding 2013), the percentage of women aged 21 to 65 who reported having had a Pap test within the last 3 years was similar in the poorest counties (75.0%) and the most affluent counties (75.2%).

**Table 2:** Prevalence of Current Smoking, Obesity, Physical Inactivity, Heavy Drinking, and Receipt of Papanicolaou Test Among Adults (Aged ≥18) in the Poorest Counties and Most Affluent Counties in Ohio, 2011–2015^a^^,^ b

Health Indicator	% (95% CI)		*P* Value^c^
	Poorest Counties	Most Affluent Counties	
Current smoking^d^	25.6 (23.8–27.5)	17.1 (15.4–18.9)	<.001
Obesity^e^	32.7 (30.8–34.5)	28.3 (26.3–30.3)	<.001
Physical inactivity^f^	29.7 (27.9–31.6)	23.0 (21.2–24.7)	<.001
Heavy drinking^g^	5.6 (4.6–6.5)	5.5 (4.4–6.6)	.92
Pap test in the last 3 years^h^	75.0 (71.4–78.6)	75.2 (70.9–79.5)	.91

Abbreviations: CI, confidence interval; Pap, Papanicolaou.

a Source of data on prevalence: Ohio Behavioral Risk Factor Surveillance System, Ohio Department of Health (13).

b Identified by the percentage of the county population living in poverty in the 2011–2015 American Community Survey (6). Twelve counties in Ohio had poverty rates of 20% or more in 2011–2015 (Adams, Ashtabula, Athens, Gallia, Highland, Jackson, Lucas, Meigs, Morgan, Pike, Scioto, and Vinton). Ten counties had poverty rates of less than 10% in 2011–2015 (Auglaize, Delaware, Geauga, Lake, Madison, Medina, Mercer, Putnam, Union, and Warren).

c Differences in percentages between poorest counties and most affluent counties determined by the 2-proportion *z* test; significance set at <.05.

d Defined as persons who reported smoking at least 100 cigarettes in their lifetime and currently smoke cigarettes every day or some days.

e Defined as a body mass index of ≥30.0 (weight in kilograms divided by height in meters squared).

f Defined as no physical activity or exercise during the past 30 days other than their regular job.

g Defined as adult men having >2 drinks per day and adult women having >1 drink per day.

h For women aged 21 to 65 years. Not collected in the 2013 Ohio Behavioral Risk Factor Surveillance System.

### Health insurance status

A significantly higher percentage of people with cancer in the poorest counties, compared with people with cancer in the most affluent counties, were uninsured (2.7% vs 1.9%), had Medicaid (8.3% vs 3.5%), had Medicare (47.7% vs 45.1%), or had military or Veterans Affairs (VA) benefits (1.6% vs 1.0%) (Table 3). The poorest counties had a significantly higher proportion of cases in which health insurance status was unknown (6.7% vs 5.7%). The most affluent counties had a significantly higher percentage of people whose primary payer at diagnosis was private insurance (42.9% vs 33.0%).

**Table 3:** Percentage of Cases by Health Insurance Status and Treatment Status for All Selected Cancers Combined in the Poorest Counties and Most Affluent Counties in Ohio, 2011–2015^a^^,^ b

Indicator	% (95% CI)		*P* Value^c^
	Poorest Counties	Most Affluent Counties	
**Insurance status**			
Insured^d^	33.0 (32.4–33.5)	42.9 (42.3–43.4)	<.001
Medicaid	8.3 (8.0–8.7)	3.5 (3.3–3.7)	<.001
Medicare	47.7 (47.1–48.3)	45.1 (44.6–45.6)	<.001
Military/Veterans Affairs	1.6 (1.4–1.7)	1.0 (0.9–1.1)	<.001
Uninsured	2.7 (2.5–2.9)	1.9 (1.8–2.0)	<.001
Unknown	6.7 (6.4–7.0)	5.7 (5.4–5.9)	<.001
**Treatment status**			
No treatment given	10.4 (10.0–10.8)	8.9 (8.6–9.2)	<.001
Treatment given	81.0 (80.5–81.5)	82.9 (82.5–83.3)	<.001
Active surveillance^e^	2.4 (2.2–2.6)	2.0 (1.9–2.2)	.006
Unknown	6.3 (6.0–6.6)	6.1 (5.9–6.4)	.55

Abbreviation: CI, confidence interval.

a Source of data on health insurance status and treatment status: Ohio Cancer Incidence Surveillance System, Ohio Department of Health (9).

b Identified by the percentage of the county population living in poverty in the 2011–2015 American Community Survey (6). Twelve counties in Ohio had poverty rates of 20% or more in 2011–2015 (Adams, Ashtabula, Athens, Gallia, Highland, Jackson, Lucas, Meigs, Morgan, Pike, Scioto, and Vinton). Ten counties had poverty rates of less than 10% in 2011–2015 (Auglaize, Delaware, Geauga, Lake, Madison, Medina, Mercer, Putnam, Union, and Warren).

c Differences in percentages between poorest counties and most affluent counties determined by the 2-proportion *z* test; significance set at <.05.

d Defined as private insurance (managed care, health maintenance organization [HMO], preferred provider organization [PPO]); fee-for-service private insurance; and insurance not otherwise specified.

e A treatment plan that involves closely watching a patient’s condition but not giving any treatment unless changes in test results show the condition is getting worse (eg, prostate cancer).

### Treatment status

A significantly smaller percentage of people were given treatment in the poorest counties (81.0%) than in the most affluent counties (82.9%) (Table 3). The percentage of cervical cancer cases in which no treatment was given was 9.6% in the poorest counties and 4.9% in the most affluent counties, although this difference was not significant. About 6% of all cancer treatment was reported as unknown in both the poorest and most affluent counties, and we found no significant difference for this variable between the 2 groups.

## Discussion

Similar to results based on US census-tract data (3), the results of our study found that cancers with the greatest disparity in incidence rates between the poorest and most affluent counties in Ohio were cancers of the cervix and larynx. Also similar to other national data, our data showed that the most affluent counties had higher incidence rates than the poorest counties for female breast cancer, melanoma of the skin, prostate cancer, and thyroid cancer. Nationally, poverty is associated with higher cancer mortality rates (1). Similarly, our study found that the poorest counties in Ohio had higher cancer mortality rates for all cancers combined, with cancers of the cervix and larynx showing the greatest disparity between the poorest and most affluent counties in Ohio in 2011–2015.

Poverty is also associated with some cancer risk factors such as tobacco use, obesity, and lack of access to cancer screening and treatment (1). Tobacco use is associated with 12 types of cancer and is estimated to cause more than 30% of all cancer deaths in the United States, including 80% of lung cancer deaths among men and women (18). Obesity is the second leading cause of preventable cancer in the United States. Overweight and obesity are associated with increased risk for developing many cancers, including adenocarcinoma of the esophagus and cancers of the breast (in postmenopausal women), colon and rectum, endometrium, kidney, liver, and pancreas (19). Higher levels of physical activity are linked to lower risks of several cancers, including colon, breast, and endometrial cancers (20). Heavy alcohol consumption is a risk factor for cancers of the oral cavity and pharynx (excluding the lips), larynx, esophagus, liver, and breast and is associated with an increased risk of cancers of the colon and rectum (21). People who use both alcohol and tobacco have a greater risk of developing cancers of the oral cavity and pharynx, larynx, and esophagus than people who use either alcohol or tobacco alone (21). Virtually all cervical cancer cases are caused by infection with human papillomavirus (HPV) (22). Women who do not regularly have Pap tests to detect abnormal cells in the cervix or tests to detect HPV are at increased risk for cervical cancer (22). Among women infected with HPV, those who smoke have twice the risk of nonsmokers of developing cervical cancer (23). Our study found that the prevalence of current tobacco smoking, obesity, and physical inactivity was significantly higher in Ohio’s poorest counties, which also had higher rates of cervical cancer and tobacco-related cancers. In addition, residents in the poorest counties in Ohio were more likely to be diagnosed at a later stage for cervical cancer, and to a lesser extent, other smoking-related cancers.

Health insurance status plays a role in cancer disparities. People who are uninsured or underinsured are less likely to have adequate cancer treatment and care. Furthermore, unequal access to screening may lead to a later stage at diagnosis and a lower chance of survival (2). In our analysis, Ohio’s poorest counties had a significantly higher percentage of people who were uninsured at the time of cancer diagnosis and were less likely to have received treatment. Other barriers to health care access may play a role in cancer health disparities. For example, all of the poorest counties, except Lucas County, are in Appalachia. These counties have fewer specialty physicians (61 per 100,000) than counties not in Appalachia (175 per 100,000) and in Ohio as a whole (155 per 100,000) (24).

This study has several limitations. First, incidence rates are affected by completeness of reporting. “Completeness” is the percentage of cancer cases diagnosed among Ohio residents that are reported to OCISS within 24 months of diagnosis. It is based on Ohio mortality rates and the SEER Program incidence to mortality rate ratio. Overall, completeness of case reporting to OCISS was an estimated 97% for 2011–2015. However, the estimated completeness of reporting for the poorest counties was 91% for all cancers combined in 2011–2015, whereas the most affluent counties had an estimated completeness of 100%. Therefore, incidence rates may be higher than indicated in the poorest counties, and higher incidence rates in the poorest counties would result in even greater differences between the 2 groups of counties. Cancer mortality rates are not affected by delayed reporting or underreporting; therefore, an analysis of cancer mortality rates may provide a more accurate comparison of the cancer burden between these 2 groups of counties. Second, BRFSS estimates have limitations. The BRFSS surveys adults living in households only. Therefore, people living in group settings such as nursing homes, military facilities, or prisons are not surveyed. In addition, adults who live in households without telephones are not included in the BRFSS sample. BRFSS prevalence estimates are based solely on respondents’ self-reported answers to survey questions. Respondents may be uncomfortable sharing private health information, or conversely, may exaggerate particular feelings or experiences, or may be tempted to provide responses that are more socially desirable. In some cases, information provided by respondents may be subject to recall bias. Finally, because it was not possible to know the poverty status and risk factors of each person with cancer in Ohio, we could not examine direct causal associations.

Our study identifies cancer disparities between Ohio’s poorest counties and most affluent counties and may help target public health interventions for cancer prevention, early detection, and control. In Ohio, the poorest counties had higher cancer incidence and mortality rates than the most affluent counties, especially for cancers of the cervix and larynx and other smoking-related cancers. Several cancers were diagnosed more often at a late stage in the poorest counties than in the most affluent counties. Targeted public health interventions such as smoking cessation programs and screening programs in poor and underserved geographic areas in Ohio may lead to a reduction in cancer disparities. Social determinants of health such as lack of health insurance and access to cancer treatment must also be addressed to reduce the burden of cancer and improve patient outcomes in this state.
